# Using Recurrent Neural Networks for Predicting Type-2 Diabetes from Genomic and Tabular Data

**DOI:** 10.3390/diagnostics12123067

**Published:** 2022-12-06

**Authors:** Parvathaneni Naga Srinivasu, Jana Shafi, T Balamurali Krishna, Canavoy Narahari Sujatha, S Phani Praveen, Muhammad Fazal Ijaz

**Affiliations:** 1 Department of Computer Science and Engineering, Prasad V. Potluri Siddhartha Institute of Technology, Vijayawada 520007, Andhra Pradesh, India; 2 Department of Computer Science, College of Arts and Science, Prince Sattam bin Abdul Aziz University, Wadi Ad-Dawasir 11991, Saudi Arabia; 3 Department of Computer Science and Engineering, Dhanekula Institute of Engineering and Technology, Vijayawada 521139, Andhra Pradesh, India; 4 Department of Electronics and Communication Engineering, Sreenidhi Institute of Science and Technology, Hyderabad 501301, Telangana, India; 5 Department of Intelligent Mechatronics Engineering, Sejong University, Seoul 05006, Republic of Korea

**Keywords:** deep learning, PIMA dataset, Type-2 diabetes, Recurrent Neural Networks, weight optimization

## Abstract

The development of genomic technology for smart diagnosis and therapies for various diseases has lately been the most demanding area for computer-aided diagnostic and treatment research. Exponential breakthroughs in artificial intelligence and machine intelligence technologies could pave the way for identifying challenges afflicting the healthcare industry. Genomics is paving the way for predicting future illnesses, including cancer, Alzheimer’s disease, and diabetes. Machine learning advancements have expedited the pace of biomedical informatics research and inspired new branches of computational biology. Furthermore, knowing gene relationships has resulted in developing more accurate models that can effectively detect patterns in vast volumes of data, making classification models important in various domains. Recurrent Neural Network models have a memory that allows them to quickly remember knowledge from previous cycles and process genetic data. The present work focuses on type 2 diabetes prediction using gene sequences derived from genomic DNA fragments through automated feature selection and feature extraction procedures for matching gene patterns with training data. The suggested model was tested using tabular data to predict type 2 diabetes based on several parameters. The performance of neural networks incorporating Recurrent Neural Network (RNN) components, Long Short-Term Memory (LSTM), and Gated Recurrent Units (GRU) was tested in this research. The model’s efficiency is assessed using the evaluation metrics such as Sensitivity, Specificity, Accuracy, F1-Score, and Mathews Correlation Coefficient (MCC). The suggested technique predicted future illnesses with fair Accuracy. Furthermore, our research showed that the suggested model could be used in real-world scenarios and that input risk variables from an end-user Android application could be kept and evaluated on a secure remote server.

## 1. Introduction

Diabetes is a metabolic disorder influenced by high blood sugar levels due to insufficient insulin release or synthesis. Diabetes was predicted to affect 285 million people globally in 2010. According to the disease’s current development pace, this figure will increase to 552 million by 2030. One in every ten people is projected to have diabetes by 2040 [1]. Diabetes is becoming more prevalent because of individual habits, divergent lifestyles, and living standards. Thus, researching how to effectively and promptly identify and treat diabetes is worthwhile. Diabetes is diagnosed based on genomic patterns. It will result in a more accurate and precise outcome and assist in adhering to better habits that are less likely to result in diabetes shortly. Very effective identification of an illness allows individuals with future illnesses to slow or postpone the disease’s development and enjoy better overall health. Machine learning techniques fall into two categories: screening future illnesses and diagnosing an abnormality [2]. Based on current and prior medical conditions, forward prediction techniques can anticipate diabetes before it occurs [3,4].

Type 2 diabetes (T2D), formerly called non-insulin-dependent diabetes) is a category of metabolic disorders distinguished through hyperglycemia, resulting in abnormalities in insulin production or insulin function. Lifestyle behaviors, including food habits, exercise, and dietary choices, may significantly affect its development. T2D is the type of disease known for decreasing the life span and reducing the standard of living. The illness may be controlled with lifestyle modification and pharmaceutical management. Thus, it is essential to have early diagnosis and treatment of T2D to help patients avoid life-threatening consequences. Many research studies have been conducted on medical diagnoses to predict illness and forecast the future with considerable efficiency accurately. Generally, most diseases are triggered by a combination of two or more gene patterns. Recognition of the combinational gene sequence by rigorous analysis of the reference genes of the healthy person with the samples that are trained genes acquired from the diseased. Deoxyribonucleic Acid (DNA) is significant for cell growth and is generally a hereditary component in each cell of an organism. As stated by A. Arshad and Y. D. Khan [5], DNA is coded through chemical bases adenine (A), guanine (G), cytosine (C), and thymine (T) that form up a cell which is unique for almost all human beings. Analysis of DNA molecular compositions of human genes is immensely used to predict illnesses associated with ancestors. The genomics study would assist in changing the lifestyle of an individual, which results in a lower risk of the disease in the future. DNA analysis can assist in the prediction of a disease that is caused by a mutation of the DNA. Biomedical engineering has recognized an enormous gene data set that could help predict various conditions. The Neural Network approach can identify gene patterns that harm cells of a human body with a high possibility of illness-causing patterns through the proposed mechanism. By incorporating Neural Networks, approaches would have high Accuracy for illness prediction with reasonably acceptable computational latency.

The advancement that has taken place in Genome-Wide Association Studies (GWAS) holds tremendous information related to various gene patterns associated with divergent illnesses that are complex and challenging to perform reductive analysis from a single locus, as stated by Cho Ys [6] and Coron [7]. The evolution of GWAS has focused on integrating data related to multi-locus across the gene that would assist in predicting complex illnesses in advance. Polygenic Risk Scores (PRS) were proposed by Duncan L. et al. [8] for risk analysis using the sum of the weight of each risk-associated locus of genomic sequence obtained from the corresponding evidence. These weights are assessed from the regression coefficient associated with each locus. These combined genetics features and correlation matrices would significantly assist the entire field of genomics study [9]. These studies on analyzing the genomic data and the tabular datasets such as PIMA would largely assist in analyzing the future illness had paved the motivation for the current study, and the role of various neural network components in the performance of the deep learning models are evaluated [10,11].

The current study is primarily motivated by the research challenges in handling genomic data and the pattern recognition for precisely identifying future illnesses. The genomic data comprises more extensive DNA sequences, which requires tremendous computational efforts to assess the disease’s probability. Earlier assessment of the future illness would assist the individual in safeguarding themselves from such disease through better living standards. Moreover, the current study has also focused on evaluating the performances of various recurrent neural network models such as RNN, GRU, and LSTM in disease prediction. Performances evaluation metrics such as the confusion matrix for Sensitivity, Specificity, F1-Score, and Mathews Correlation Coefficient measures are considered in the current study. 

The recurrent neural network components such as RNN, GRU, and LSTM are efficient in learning from past experiments and can simultaneously process a sequence of inputs and outputs, which is a kind of sequence-to-sequence network that is exceptionally efficient in handling genomic data. The RNN-based neural network may represent a set of records such that each pattern is thought to rely on preceding ones. The Hidden State, which remembers certain information about a sequence, is the core and most essential aspect of RNN. LSTM feeds genetic sequences into a network and makes assumptions based on the sequence data’s discrete time steps. It can learn long-term dependencies, which is notably valuable for sequence prediction issues. GRU uses less memory and is comparatively faster than the RNN and LSTM models. But can effectively work with smaller sequences. GRU employs fewer training parameters, requires less memory, executes quicker, and learns faster than LSTM, although LSTM is much more accurate on more extended sequence datasets.

The main contributions of this work are as follows:

- The reference gene sequence is analyzed against the trained genomic data for possible gene pattern matching. As well, the further correlation between the reference gene and gene pattern associated with diabetes is assessed.
- The probabilistic estimations are performed by the softmax layer towards the future illness based on the gene correlation. Additionally, based on the probabilities, the risk factor outcome is yielded.
- The proposed RNN model is evaluated over the tabular patient data such as PIMA for risk analysis, where the auxiliary memory components such as GRU and LSTM are integrated for better prediction performance.
- The feature selection and weight optimizations are performed over the features of the PIMA dataset for better prediction outcomes.
- The outcome of the present study is being evaluated against conventional classification techniques such as Decision Tree, J48, K Nearest Neighborhood, Logistic Regression, Naive Bayes, Random Forest, and Support Vector Machine.

The entire paper is arranged as follows. The paper’s first section introduces the proposed approach and the Genomic domain. Section 2 presents the literature review of existing studies focusing on various disease prediction techniques using genomic and tabular data. Section 3 presents the methodology of the proposed model where various aspects such as background work of the domain that highlights data collection, data preprocessing, feature extraction strategies, and RNN with various memory components are presented. Section 4 presents the result and discussion. Section 5 presents the conclusion and future scope of the proposed model.

## 2. Literature Review

### 2.1. ML Models for Smart Diagnosis of Type-2 Diabetes 

Machine Learning is the most emerging technology for addressing inevitable problems in various domains. Machine Learning through supervised, semi-supervised approaches, or weakly supervised approaches, is used with data from various sources, including medical records and information obtained from wearable gadgets, to forecast an illness. In either of these approaches, sickness cannot be predicted much earlier, and the patient cannot get rid of the illness by changing his or her lifestyle in a short period. The polygenic scores-based approach is among the most predominantly used strategies for the earlier prediction of an illness. The Polygenic Score approach has been tremendously evaluated before it is used in clinical trials. It is also used in illness screening mechanisms, as in the study of So et al. [12]. The current research and the genomic analysis could change lifestyles and reduce illnesses such as heart attack, cardiovascular diseases, cancer, and Alzheimer’s disease. The process of polygenic risk score involves two significant phases: discovery and validation. The Discovery Phase identifies risks through a statistical association test using either linear or Logistic Regression. The later phase validates approximations performed in the earlier stage for extracting information related to Single Nucleotide Polymorphism (SNP).

Deep learning (DL) [13,14] is the field of Machine Learning that is extensively used in predicting type-2 disease by processing the blood glucose level and spectrogram images generated from the blood glucose levels. Moreover, the DL models could also be used with tabular datasets such as PIMA for the prediction of diabetes. Every layer in the DL model reflects a degree of acquired information. The layer closest to the input layer reflects low-level data elements, whereas the layer closest to the output layer shows a higher degree of discrimination with more concise notions. Deep learning generally needs more data for precise classification and also needs tremendous computational resources for processing [15]. The major limitation of the deep learning models is that the decision mechanism is not interpretable, which limits the trustworthiness of the models. 

Clustering is one of the most predominantly performed operations with un-supervisory approaches using dimensionality reduction approaches such as Singular Value Decomposition (SVD) [16], Principle Component Analysis (PCA) stated by Konishi T. et al. [17], Apriori stated by S. Mallik et al. [18]. Dynamic thresholding-based FP-Growth was stated by Mallik S. et al. [19] for treating unusual illnesses and certain types of diseases with unknown variations with different symptoms. However, most of these approaches do not label the output data, as the provided input does not have any labels. The Accuracy of the un-supervisory method is a significant concern as classes are not marked. In some instances, the proposed algorithm might end up misinterpretation. A classification-based illness prediction is a supervisory approach that includes various mechanisms such as Linear and Polynomial Regression, Decision Tree, Random Forest, and many other systems, including the Support Vector Machine (SVM) used by Huang S. et al. [20], K-Nearest Neighbour used by Parry R. et al. [21], and Logistic Regression approach that exhibits better efficiency in terms of accuracy and precision other classification models. Supervisory approaches exhibit optimal performance for known cases. The Accuracy of the prediction outcome is directly proportional to the training set size, which needs many computational efforts. However, in some cases, the approach diverges due to excessive training.

Random Forest is a rapid implementation approach using the Ranger package in the R tool described by Wright and Ziegler [22], which is used to predict future illness from tabular data such as PIMA. Artificial Neural Networks based on illness prediction mechanisms, as discussed by Anifat O. et al. [23] and Mantzaris D. et al. [24] involve a more profound architecture that includes input and output layers alongside multiple hidden layers to process records iteratively, moving data among layers that would minimize the loss function and acquaint weights and biases of each layer. Various ensemble approaches, such as random forest and boosting, have been experimented with as alternatives to machine learning approaches for predicting future illness. Exponential research has been conducted using either of those approaches with real-time and simulated data. The ensemble approaches work faster for classification when compared to the conventional classification models. However, either of the models ends up with non-additive issues. The resultant effect in the forward direction of the layers would determine the predictive analysis, and the backward pass would assess the standard error among the prediction made and the ground facts.

### 2.2. Deep Learning for Type-2 Diabetes

The Deep Learning (DL) model implements the framework that infers target gene expression obtained from the expression of landmark genes. Utilizing 111,000 individual gene patterns over a Gene expression Omnibus2, Deep Neural Network-based Gene Analysis model (D-GEX) trained a feedforward neural network through three hidden layers. DL models outperform linear Regression in summarizing expression levels of over 21,000 human genes based on a collection of landmark genes with about 1000 sequences. Although the DL model is more accurate than conventional classification models, performance is not adequate in the healthcare domain, where the design of DL models needs to be improved. The deepVariant model outperforms all other recent neural network models [25]. DeepVariant generalizes its training samples by utilizing various human genome expressions as train and test datasets.

Additionally, when training with human gene expressions and evaluating with a mouse genomic expressions dataset, DeepVariant obtained Accuracy that outperformed training with mouse data. DeepFIGV is a deep learning algorithm that uses DNA sequences to predict locus-specific signals from epigenetic tests. DeepFIGV quantifies epigenetic variance by employing several investigations with similar cell patterns and experiments [26]. It combines the entire gene sequence to provide a customized genetic line for each person. The Gene Co-Expression model is a differential network analysis model extensively used in gene data analysis to identify gene sequence similarities and topologies [27]. This model considers two classes of the gene through which the classification model is implemented. However, the Gene Co-Expression model has to deal with comparatively larger features than the size of the data and the non-linearity of the network architecture, where dependencies would make it difficult to trust the model’s predictions. Reinforcement Learning (RL) based intelligent systems such as Q-Learning, State Action Reward State Action (SARSA), Deep Deterministic Policy Gradient (DDPG), and Deep Q Network (DQN), as stated by Travnik Jaden B. et al. [28] are the most suitable for handling healthcare to better forecast a future illness with minimal training of the algorithm recovers. The underlying technology remains the same with minimal training. The algorithm is mechanized to learn from its previous experiences.

Various studies have been presented to predict future illness through existing patient data using machine learning algorithms. Predicting future illness has become a demanding topic in healthcare [29]. Several studies have used machine intelligence techniques to analyze the Pima Indian Diabetes Dataset. C. Yue [30] has investigated various hybrid approaches, including Neural Networks, integrated Quantum Particle Swarm Optimization (QPSO), and Weighted Least Square (WLS) Support Vector Machine (SVM) for diabetes prediction, with the WLS-SVM hybrid model showing a classification accuracy of 82.18%. However, the hybridization model needs considerable effort in the evaluation process. In addition, the SVM model is not suitable for working with larger data [31]. Moreover, the SVM model underperforms if the number of attributes for every data point exceeds the training samples. The combinational models for diabetes prediction using Cross-validation and Self-Organizing Maps (SOM) have achieved an accuracy of 78.4% [32,33]. SOM can rely on the associated weights of neurons for precise classification. Inappropriate assignment of initial weights may impact the model’s performance. A C4.5 technique [34] has been used to analyze the PIMA dataset, attaining an Accuracy of 71.1%. The model works through the entropy value associated with the feature vector. The conventional classification models exhibit poor performance when working with distinct feature vectors [35]. 

A fuzzy entropy approach for feature selection for a similarity classifier has been evaluated against various medical datasets, such as Pima–Indian diabetes, exhibiting an accuracy of 75.29% [36]. A fuzzy model primarily depends on the membership evaluation that requires considerable effort. Non-linearity in evaluating the model will limit the model’s performance [37]. Genetic Algorithm (GA) with Radial Basis Function Neural Network (RBF NN) has been used in the evaluation process of diabetes data, exhibiting an accuracy of 77.39% over the testing dataset [38]. Moreover, for artificial evolutionary algorithms such as GA, the most prohibitive and restricting element is frequently repeated fitness function assessment for complex gene patterns. Hybridization of models with GA would need more computational efforts than neural networks alone. Various cutting-edge technologies for the classification and prediction of type-2 diabetes are presented in Table 1.

All the mentioned models rely on tabular datasets such as PIMA and ECG signals [47] in classifying the records with possible diabetic illnesses. The current study considers that genomic data yields a better patient-centric outcome than tabular data.

### 2.3. Genomics for Type 2 Diabetes

Many research studies have been carried out on genetic-based illness prediction. Incorporating machine learning approaches with genetic-based illness prediction could result in an accurate outcome. This has intensified the role of Artificial Intelligence (AI) in healthcare. It has been estimated that approximately $36 billion will be invested in AI by 2025 [48]. Deep genomics through machine learning approaches has outperformed accuracy in predicting and diagnosing illnesses such as cancer with minimal inclusion of radiologists. It is desired to have sufficient biological knowledge to understand how genetics can help us predict various conditions and analyze each chromosome to identify the disease-causing gene. Pre-existing research studies have focused on genomics and gene interaction patterns of various persistent illnesses such as Alzheimer’s, multiple cancers, and Parkinson’s.

**Figure 1:**
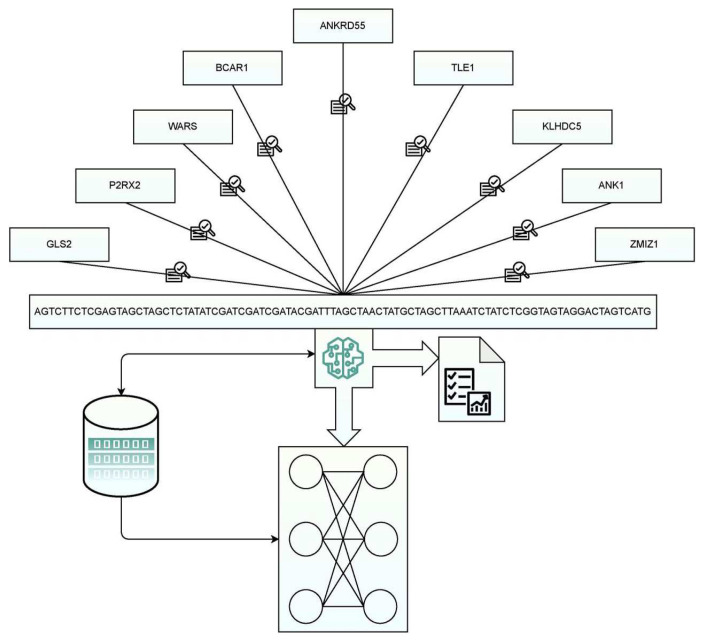
Gene analysis-based disease perdition framework.

Many aspects need to be considered in the predictive analysis of an illness, as a gene mutation might lead to two or three diseases. The main challenge when handling genomic data for illness prediction is that the prototypical microarray image consists of fewer records. In contrast, the number of fields concerning genres could result in a few lakhs that might misinterpret the data with a significant false-positive ratio. Enhanced Gene-Set analysis can be deployed to extract and analyze genes resulting in soaring throughput on molecular assessments. Gene-Set analysis, as stated by Mooney M. A. and Wilmot B. [49] and Mathur R. et al. [50], is also referred to as pathway analysis, is meticulous in aggregating gene-sets with identical properties or sequences per the reference’s gene trained or presented in the disease’s knowledge base. Genome-wide association studies (GWAS) have demonstrated that many disease-causing genes are related to human diseases. GWAS has also provided polygenic characteristics of diseases. Figure 1 presents a block of GWAS in disease prediction. There are many steps during a gene-set analysis. They are shown below as Steps 1 through Step 6:

Step 1: Preliminary genome-wide analysis and data preprocessing;
Step 2: Identifying gene-set definitions whose patterns have to be recognized;
Step 3: Processing genomic data such as filtering and identifying gene patterns;
Step 4: Identify gene set analysis models, such as identifying the statistical hypothesis;
Step 5: Assessing the statistical magnitude;
Step 6: Report summarization and visualization.

Gene data include metadata about the information associated with type 2 diabetes, consisting of alleles, MegaBase, and Single nucleotide polymorphisms. An allele is a word that denotes a particular gene sequence copy associated with a specific context. A mutation might be considered one of two or more varieties of a particular gene. Most individuals have SNPs. However, some variants are more prevalent than others in particular populations. A single DNA-building unit, the nucleotide, is found at tens of thousands of different sites on the human genome. In genetics, a MegaBase is a unit of length representing a genomic region’s length. MegaBase is used to determine the distance between two genes. Values of these gene features mentioned above are considered when evaluating the possibility of feature disease.

A highly dense genotyping collection is considered for coverage throughout the whole genome, such as covering common and uncommon variations in the genome. These gene sequences contain many single-nucleotide polymorphisms (SNP) that can significantly improve the capture of low-frequency variations, which is advantageous to users of other genome-wide collections. Gene sequences that hold a higher possibility of T2D in the future are listed in Table 2. Disease-corresponding gene sequences are cross-validated against individual data for forecasting the likelihood of the disease.

## 3. Methodology

This study is focused on predicting future illnesses such as type-2 diabetes from genomic and tabular data. Genomic data are analyzed for possible gene expression highly likely to be affected by type-2 diabetes. Tabular data from the PIMA dataset with various features are also explored through the proposed RNN model by identifying the feature vector’s pivotal features. The proposed model relies on the Deep Neural Networks (DNN) framework for analyzing the genomic data, making the precise assessment of possible future illnesses with better Accuracy than the conventional pattern-matching techniques. DNN is a probabilistic measure that would summarize the possible illness outcome that would better assist in decision-making by the physicians. The working procedure and implementation details are discussed in the current section. The models are trained from the available gene base from scratch initially, and at the later stages, the model learns from the experimental outcomes.

### 3.1. Recurrent Neural Network Model for Type 2 Diabetes Forecasting Based on Genomic Data

Predictions of future illness can be performed through Convolutional Neural Networks (CNN), as stated by Leevy J.L. et al. [51] and Yadav S.S. and Jadhav S. M. [52] using Recurrent Neural Network (RNN) module-based architecture described by SivaSai J.G. et al. [53]. CNN model consists of many intermediate nodes connected. Each node is significant in delivering the output following the anticipated outcome. RNN is robust in handling variable-length input sequences with the help of internal auxiliary memory modules [54]. The detailed architecture along with the implementation procedure for the proposed approach, is presented in this section.

With the proposed approach, gene patterns are analyzed against pre-trained sequences of genes that cause the disease. For the effective implementation of illness prediction, the recurrent neural network component is incorporated with gene set analysis, which could minimize the false positive ratio. The Recurrent neural network model is a layered architecture approach where each layer works independently. The output of the previous phase is fed as the input for the next phase. Recurrent neural networks can transform individualistic components into contingent components by adjusting each layer’s weight and bias by minimizing the number of parameters to be considered and reducing the complexity of memorizing the previous layer’s output. The responsibility of each layer is presented in this section, along with the working procedure of the proposed model.

#### 3.1.1. Data Collection and Processing

Gene-related data were acquired from the open-access comprehensive miRbase-18.0 R dataset with human gene sequences of 10,094 records labeled and annotated [55,56]. In the present experimental study, 303 samples were considered for the training and validation of the model at 70:30 proportion, respectively. Generally, gene sequences are 84 nucleotides in length, ranging from 43 nucleotides to 154 nucleotides.

The data acquired from online repositories must be processed following the model’s outcome. The information is organized in tables to be further refined to predict gene sequence better, including aligning the region of interest in genomic patterns. Gene sequences could be expressed as a grid in which each location corresponds to a single-hot vector containing letters A, C, G, and T. Gene expression is indeed a matrix containing absolute values, each such element representing the pattern that is an integral part of the gene in a particular environment, such as a cell. Spatial information is often described as a three-dimensional array, with two dimensions representing the entity’s actual location and a third dimension representing colors or genes. Typically, texts are defined as a one-hot matrix for each token entering a stable database. While most cells have the same genome, individual genes are expressed at highly variable amounts in variable tissues and cells in response to various treatments and settings. Such degrees of gene expression could be quantified by measuring levels of mRNA transcripts. In such context, comparison of gene expression of patients with the illness to that in healthy cohorts (without the disease of interest) and different link genes with the diseases underlying biological systems.

#### 3.1.2. Feature Selection

When analyzing gene data for illness prediction, features are significant in obtaining an accurate and precise outcome. Feature selection is one of the vital phases of the proposed approach. The feature selection process performed during the training step would have a noticeable contribution to the dimensionality reduction of gene data, including discarding irrelevant data and recognizing vital records in the dataset. The proposed approach’s performance depends on the feature selection mechanism in the present work. It is significant in identifying the diseased gene from the extracted genomic information for the human body. Minimum Redundancy Maximum Relevance (mRMR), as stated by Zena M. Hira and Duncan F. Gillies [57] and M. B. Shirzad and M. R. Keyvanpour [58], was used for feature selection and extraction of microarray data in the current study.

The minimum Redundancy Maximum Relevance (mRMR) approach maximizes the relevancy of components concerning the genomic information and minimizes the number of corresponding classes. mRMR-based feature selection technique that favors features that have a strong correlation with class but a low correlation among themselves. In the feature extraction process, divergent statistical metrics are considered, including Mutual Information (MI), which assesses the entropy of a random variable concerning other variables in the corresponding class. The mRMR approach can also be used with both continuous and discrete variables. The amount of MI among features is used to calculate redundancy. If the value of MI is substantial, it indicates a significant degree of data redundancy among the two characteristics, i.e., redundancies. A lower redundancy measure value suggests more effective feature selection criteria. The purpose of redundancy is to locate the feature with the lowest MI value among all features. According to the premise that the lower the value of information redundancy across features, the more helpful it is to activity categorization, which may be stated by decreasing MI among features [59]. The following equation determines the gene that is not redundant for a set of features $\beta \left(x\in \left\{1,2,\ldots f\right\}\right)$

(1) $$M_{r}=\frac{1}{{\left|f\right|}^{2}}\sum_{\alpha ,\beta \in C}\mathrm{MI}\left(\alpha ,\beta \right)$$

In the above Equation (1), the discrete variable MI is the Mutual Information, variables α and β represent genes, and |f| represents the number of features in class C. The maximum relevance concerning the target class is determined through the following equation:

(2) $$R=\frac{1}{\left|f\right|}\sum_{\alpha \in C}\mathrm{MI}\left(\gamma ,\alpha \right)$$

In the above Equation (2), the variable γ is the class label for discrete variables. F-Statistics for assessing the mean of two classes are significantly divergent for determining the maximum relevance among corresponding genes and the class label. The minimal redundancy that approximates the correlation of the complementary gene pairs in the class is approximated as shown in Equations (3) and (4)

(3) $$R=\frac{1}{\left|f\right|}\sum_{\alpha \in C}F_{s}\left(\alpha ,\gamma \right)$$

(4) $$M_{r}=\frac{1}{{\left|f\right|}^{2}}\sum_{\alpha ,\beta \in C}x\left(\alpha ,\beta \right)$$

The mutual information among the two gene sequences let them be p and q, and if some gene-sequence of p is there in the gene sequence q. The *MI* for the gene sequences is assessed using the generic formula for mutual information, as shown in Equation (5).

(5) $$MI\left(p;q\right)=\sum_{p,q}f\left(p,q\right)log\frac{f\left(p,q\right)}{f\left(p\right),f\left(q\right)}$$

**Figure 2:**
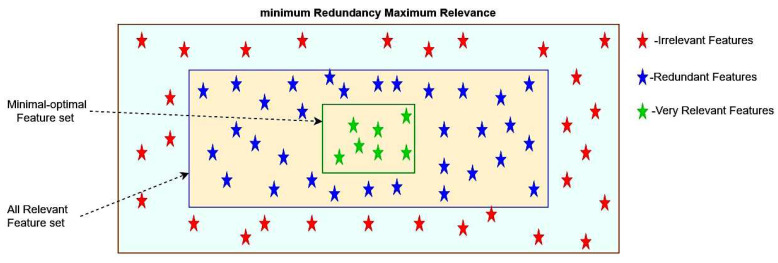
Diagram representing the mRMR Features selection technique.

The Pymrmre package helps work with the mRMR method by employing an ensemble mechanism to further investigate the feature map and construct a more robust feature set. Figure 2 represents the feature selection mechanism for selecting the optimal features for gene-data analysis.

#### 3.1.3. Layered Architecture of RNN-Based Prediction Model

There are multiple layers in the proposed RNN model. Each plays a vital role in performing the predictive analysis of the illness, according to Carrara, F., Elias [60], and Che et al. [61]. The RNN model has kernels that work on inputs to create a feature map to detect referee patterns in the corresponding input sequence. The outermost layers would be the input and output layers. There are many other intermediate layers, including the Convolutional, max-pooling, Flattening, fully connected, and softmax layers. The outermost layer captures the gene sequences that must be validated against the training set. The inner Convolutional layers are used to handle complex patterns. Each of those Convolutional layers also decomposes gene sequences.

The pooling layer acts as the interface between two convolution layers. Its focus is on minimizing the number of parameters required for processing the data, thereby handling overfitting. The pooling layer is responsible for reducing the spatial size of the model so that the model is computationally feasible. The max-pooling layer would result in the statistical outcome of decomposing the input to the minor extent possible and performing components’ filtering. Members that hold the maximum values are processed to the further stage. The rest of the components are left unprocessed. To flatten the layer associated with the conversion process of the data obtained from the previous layer, it is necessary to create a one-dimensional array of gene data that contain data to be fed to the next layer. The convolutional result is flattened to compress the outcome of convolutional layers into a single lengthy feature vector. The final classification model is termed a fully connected layer. It is linked to the output. The fully connected layer does have connections to all nodes in the layer. It is feasible to learn all nonlinear combinations of various complex patterns. The reasonability of this layer is to obtain the probability of the gene causing the abnormality. The fully connected layers comprise two significant layers. The first fully connected layer gets the input data from parameter analysis and labels the input GENE sequence for accurate prediction through weights. The fully connected output layer approximates probabilities of illness-causing genes from gene sequences [62].

**Figure 3:**
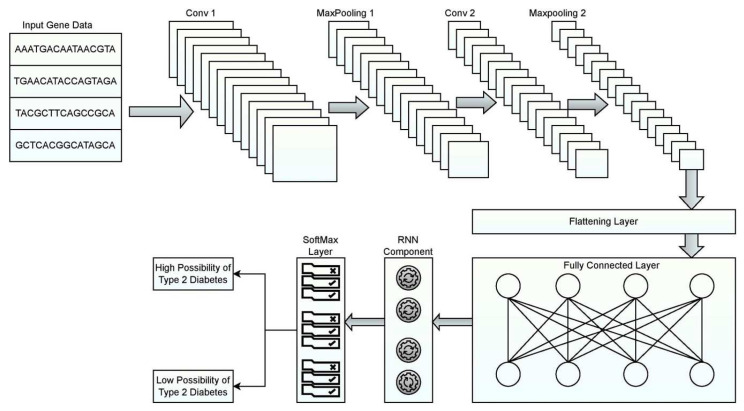
Layered architecture diagram of the RNN model.

In addition, the Softmax layer expands the concept into something similar to a multi-class environment. Specifically, in a multi-class classification issue such as disease predictions, Softmax gives decimal probability to each class. The sum of all such probabilities associated with each category is equivalent to 1.0 in the long run when dealing with decimal probability. Figure 3 presents the layered approach of RNN used in the prediction model.

Convolution is an operation that transforms a function into an output component; it is a technique that follows a certain sequence and involves intertwining two different information sources. Every single Convolutional Neural Network starts with a Convolutional Layer as its very first layer. The input is subjected to a convolutional operation in convolutional layers, and the output is then passed on to the next layer. A convolution reduces the values of all the pixels included within its receptive field to one. ReLU activation function is used with the Convolution layer. When using ReLU, all of the negative pixels are converted to 0 via an element-wise procedure. The result is a corrected feature map, which adds non-linearity to the network.

- After the Convolutional layer, the pooling layer is often applied. The pooling layer’s purpose is to minimize the volume of the input matrix for subsequent layers. In the current study, the MaxPooling function is used in the current study.
- A flattening operation transforms data into a one-dimensional array to be used in a subsequent layer. This is conducted so that CNN’s output may be sent to a fully connected network.
- A neural network is a collection of non-linear, mutually dependent functions. Neurons are the building blocks of every single function (or a perceptron). The neuron uses a weights matrix as a fully connected layer to apply a transformation matrix to the input vector. The result is then subjected to a non-linear transformation via a non-linear input signal s as shown in Equation (6).
  (6) $$f_{c}=f\left(\sum_{i=1}^{p}\omega_{ck}a_{i}+\omega_{c0}\right)$$
- One way to represent a set of numbers as probabilities are to use the Softmax mathematical function, which multiplies all the values in a set by the scale at which they appear in the vector. The likelihood of belonging to each class is calculated using the outcome of the softmax algorithm.

#### 3.1.4. RNN Component Structure

A recurrent Neural Network, also known as a back-feeding neural network, is a more robust alternative to conventional feedforward neural networks as it does not need an internal auxiliary memory. As the outcome of a current input relies on the previous calculation, RNN is recurrent. After the outcome has been produced, copying and sending the output into the recurrent network is known as “back-feeding.” The decision-making process analyses what it has learned from the prior information and applies it to the present situation. Using the gene patterns present in the sequence, RNN may extract the correlated patterns that result in type-2 diabetes. The same could be employed in analyzing variable-length gene data for the probability of being affected by type 2 diabetes.

An RNN can evaluate any sequences, irrespective of length, iteratively through its transition function over the state vector $O_{i}$. At iteration i, state activation may be calculated as a function of the input sequence character $Z_{i}$ and the prior state vector $N_{i-1}$ transformed into the $R_{i}$ in the current state cell. The tanh is the activation function associated with each cell. In RNN, the vanishing gradient issue is considered the most crucial challenge. More extensive sequences need an activation function such as tanh with a high second derivative that can maintain the gradient over iterations. Mathematical notations for each RNN module are presented in Equations (7)–(11).

(7) $$N_{i}=\sigma_{N}\left(x_{i}\right)$$

(8) $$\sigma_{N}\left(x_{i}\right)=\sigma_{N}\left(\alpha_{N}z_{i}+\beta_{N}R_{i-1}+b_{N}\right)$$

(9) $$g_{i}=\sigma_{g}\left(x_{t}\right)$$

(10) $$\sigma_{g}\left(x_{t}\right)=\sigma_{g}\left(W_{g}N_{i}+b_{N}\right)$$

(11) $$O_{i}=\tan h\;\left(W_{g}O_{i-1}+W_{g-1}Z_{i}\right)$$

**Figure 4:**
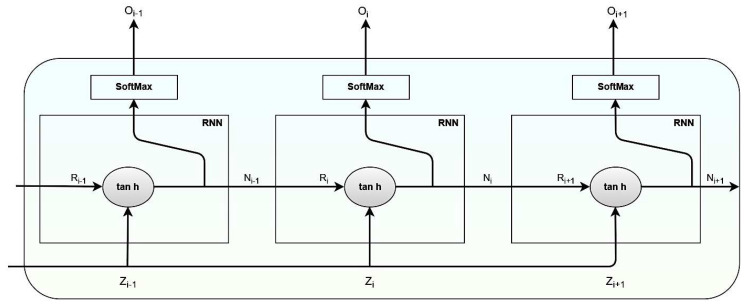
Image representing the RNN cell structure.

In Equations (7)–(11), the variable $R_{i}$ denotes the input vector of size $\left(1\times \;x\right)$, the variable${\;Z}_{i}.$ The input for the RNN cell denotes the input vector of length $\left(1\times \;x\right)$. Variables α and β denote the parameter matrix associated with pivotal features. The bias is represented by $b_{N}$. Variables $\sigma_{N}$ and $\sigma_{g}$ denote the activation function in the RNN cell. Variable $W_{g}\;$ and ${\;W}_{g-1}$ denote weights associated with the cell in the current and previous state. The RNN cell structure is presented in Figure 4, where the output of the previous component is fed as the input for the upcoming component in the RNN cell.

In the RNN model, the tan h denotes the Activation function, which implements a non-linearity that negates maximum activation values, creating a negative activation −1. The softmax layer deliberates probabilities that will assist in determining the possibility of future illness from the provided input genomic data.

#### 3.1.5. GRU Component Structure

GRU’s component in neural networks is used to address the degradation issue and create a feasible deeper layout for better Accuracy that can retain lengthy semantic patterns without calibrating model parameters [63]. The GRU component consists of the update gate and the reset gate. The update gate regulates the inflow of data to the memory component. The reset gate regulates data flowing out of the memory component, GRU. The gating unit controls the data flow inside rather than having a separate memory component to perform the task. The unit consists of two activation functions: σ and tanh. The output of the current units is identified by $cs_{t}$ becomes the input for next unit as $cs_{t-1}\;$over the time t. The variable $\alpha_{t}\;$ is assumed as the input training data and $\beta_{t}\;$ is the corresponding output generated by the activation functions ${\;\Gamma }_{r}$ and ${\;\Gamma }_{u}\;$ that denotes the reset gate and the update gate, respectively. The value of ${\;\Gamma }_{u\;}$ lies in between 0 and 1. When its values are close to 0, more data from the previous states are retained. The range of the variable $\Gamma_{r}\;$ lies in between −1 and 1. When the value is close to −1, it implies that more previous data are ignored. The GRU can be shown mathematically through Equations (12)–(15).

(12) $$\Gamma_{u}=\sigma \left(\omega_{u}\left[cs_{t-1},\alpha_{t}\right]+bias_{u}\right)$$

(13) $$\Gamma_{r}=\sigma \left(\omega_{r}\left[cs_{t-1},\alpha_{t}\right]+bias_{r}\right)$$

(14) $${\hat{cs}}_{t}=\tan h\left(\omega_{cs}\left[\Gamma_{r}\times cs_{t-1},\;\alpha_{t}\right]+bias_{cs}\right)$$

(15) $$cs_{t}=\left(1-\Gamma_{u}\right)\times cs_{t-1}+{\;\Gamma }_{u}\times {\hat{cs}}_{t}$$

**Figure 5:**
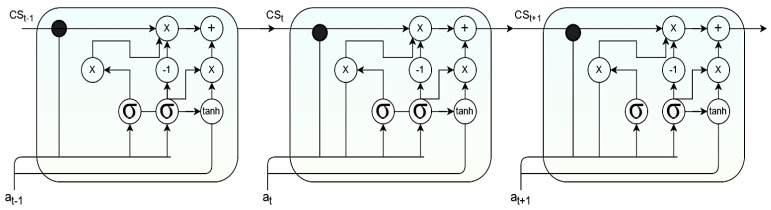
Image representing the GRU cell structure.

From Equations (12)–(15), variables $\omega_{u}$, $\omega_{r}$, and $\omega_{cs}$ designate weights associated with training the update gate, reset gate, and candidate activation, respectively. Similarly, variables $bias_{u}$, $bias_{r}$, and $bias_{cs}\;$ designate the bias associated with the update gate, reset gate, and candidate activation, respectively. Figure 5 presents the architecture of the GRU module.

#### 3.1.6. LSTM Component Structure

LSTM component is often used in recurrent neural network designs for pattern estimation issues in the sequential data over divergent time scales. Memory cells handle memory components in an abstract LSTM layer module, including an input and output gate, a forgetting gate, and a window connection [64,65]. Associated weights are comparable to those that change during a model’s training process to regulate input and hidden states. Activation functions for the LSTM component are explained with Equations (16)–(20). States are identified through variable $S_{t}$ with a hidden state vector identified by $\vartheta_{t}$ concerning the time  t over the input $i^{t}$.

(16) $$Input\;Gate\;\left(\rho_{t}\right)=\sigma \left(i^{t}\omega_{i\rho }+\gamma_{t-1}\omega_{\gamma \rho 0}+ps_{t-1}\omega_{ps\rho }+bias_{\rho }\right)$$

(17) $$Output\;Gate\;\left({ο}_{t}\right)=\sigma \left(i^{t}\omega_{iο}+\gamma_{t-1}\omega_{\gamma ο}+ps_{t}\omega_{psο}+bias_{ο}\right)$$

(18) $$Forget\;Gate\;\left(\chi_{t}\right)=\sigma \left(i^{t}\omega_{i\chi }+\gamma_{t-1}\omega_{\gamma \chi }+ps_{t}\omega_{ps\chi }+bias_{\chi }\right)$$

(19) $$Cell\;State\;Gate\;\left(ps_{t}\right)=\chi_{t}\cdot ps_{t-1}+\rho_{t}\cdot \tan\gamma \left(i^{t}\omega_{ips}+\gamma_{t-1}\omega_{\gamma ps}+bias_{ps}\right)$$

(20) $$LSTM\;Output\left(\gamma_{t}\right)={ο}_{t}\cdot \tan\gamma \;(ps_{t-1})$$

**Figure 6:**
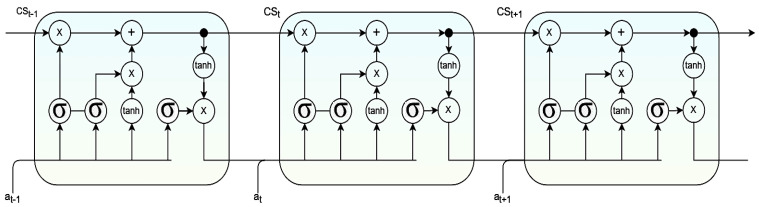
Image representing the LSTM cell structure.

From Equations (16)–(20), variables $\omega_{i\rho }$, $\omega_{iο}$, $\omega_{i\chi }$, and $\omega_{ips}$ designate weights associated with the input, output, forget, and cell state gates, respectively. In addition, $\omega_{\gamma \rho }$, $\omega_{\gamma ο}$, and $\omega_{\gamma \chi }$ designate weights associated with the hidden layer. Similarly, variables $bias_{\rho }$, $bias_{ο}$, $bias_{\chi }$, and $bias_{ps}$ designate the bias component associated with the input gate, output gate, forget gate, and cell state gate, respectively. The architecture of the LSTM component is presented in Figure 6.

#### 3.1.7. Working Procedure of the Proposed Approach

The working procedure presents the sequence of tasks performed for future illness prediction, including tasks ranging from initial data acquisition to final assumptions of the future illness.

Step 1: Acquire gene data from the annotated miRbase data set;
Step 2: Data are preprocessed to remove the outlier data and fill out acquired data gaps;
Step 3: Data is converted into 1D data, followed by aligning of genomic patterns;
Step 4: Data is categorized into a training set (80% of the data) and a testing set (20% of the data);
Step 5: Patterns are labeled based on sequence patterns of various illnesses. Moreover, weights are assigned in the later phases according to the correlation between the input sequence and the trained gene pattern;
Step 6: When a new GENE sequence is fed as input for testing the algorithm, features are extracted through the mRMR approach that is pivotal in the prediction process;
Step 7: The cumulative weight is evaluated from assigned weights based on the correlation of gene sequences between the input and the trained set;
Step 8: Based on the approximated weight of the gene sequence, the probability of a future illness is assessed;
Step 9: Final assumptions are made based on probabilistic approximations.

### 3.2. RNN Model for Illness Prediction from Tabular Data (PIMA Dataset)

The possibility of being affected by a chronic disease such as type-2 diabetes is analyzed from the tabular data with various features such as Stabilized Glucose, age, High-Density Lipoproteins (HDL) Ratio, Total Cholesterol, First Systolic Blood Pressure, Second Diastolic Blood Pressure, body mass, the height of individual, gender, and many other things. Significant features are selected, weights are adjusted in favor of pivotal features, and prediction is performed based on the feature vector. The significance of these features in the evaluation process has been discussed in earlier studies on a similar feature vector for type 2 diabetes [66,67,68]. Ranks associated with each of these features are presented in Table 3, shown below.

#### 3.2.1. Feature Weight Initialization

Features are essentially important for analyzing the possibility of diabetes disease. Weights associated with features and corresponding layers are updated over iterations [69]. When the feature is significant, it will cascade forward via hidden nodes, showing greater influence over output nodes. Thus, weighting such a significant feature is important. The feature that contributes more to the prediction process will be given considerable weightage for further processing. After training, the feature weight is obtained from the trained neural network, as shown in Equation (21).

(21) $$I_{w}=\sum_{i=0}^{p-1}\sum_{j=0}^{q-1}\left|\omega_{i,j}\times \omega_{j,k}\right|$$

From Equation (21), the variable ${\;I}_{w}$ is the initial weight assigned to the feature vector, $\omega_{i,j}$ denotes the network weight between the input node i through hidden node j. Similarly, the variable $\omega_{j,k}$ denotes weights of the hidden node j through output node k. The summation covers all potential forwarding routes between input node i and output nodes. For a few less significant features in the evaluation process, their weights are adjusted so that the sum of approximated weights of less significant features is equivalent to the total number of features. Associated weights for less significant features are given through Equation (22).

(22) $$\omega_{\mathrm{lsf}}=\frac{1}{n}\sum_{i=0}^{n-1}\omega_{\mathrm{lsf}}$$

From Equation (22), the variable $\omega_{\mathrm{lsf}}$ designates less significant weights, and the variable n designates the number of features in the considered problem. In the current context, the value of n is 8 from the Pima dataset.

#### 3.2.2. Weight Optimization

Weights associated with features must be optimized regularly for better performance of the model. These weights are optimized concerning the loss function and model parameters associated with each parameter in the training dataset [70,71]. In the current study, the input-target pair $\left(i,j\right)$ and the $\left\{\left(i_{p},j_{p}\right),0\leq p\leq n\right\}$ denote the training set. The validation set is associated with the model for fine-tuning the model’s performance using the set $\left\{{i^{ʹ}}_{p},{j^{ʹ}}_{p}),0\leq p\leq m\right\}$, where the size of m is much smaller than the size of records in n. The RNN model is denoted by $ℜ\left(p,\theta \right).$ The associated loss function will be $L\left(j^{\prime },j\right)\;$which is desired to be minimal, where $j^{\prime }=ℜ\left(i,\theta \right)$. The expected loss associated with the training set is determined through the variable $T_{l}$ as shown in Equation (23).

(23) $$T_{l}=\frac{1}{n}\sum_{p=0}^{n-1}L\left(j^{\prime },j\right)$$

(24) $$T_{l}=\frac{1}{n}\sum_{p=0}^{n-1}f_{p}\left(\theta \right)\;\mathrm{where}\;L\left(j^{\prime },j\right)=f_{p}\left(\theta \right)\;$$

In Equation (24), the function $f_{p}\;\left(\theta \right)$ is the loss function concerning data $i_{p}$. Weights associated with parameters are optimized to minimize the weighted loss through Equation (25).

(25) $$O{\left(\theta \right)}_{w}=\theta^{\prime }\sum_{p=0}^{n-1}\omega_{p}f_{p}\left(\theta \right)$$

The value of the variable $\omega_{p}$ is not known at the initial iteration. The value ${\left\{\omega_{p}\right\}}_{p=0}^{n-1}$ is tuned by training hyperparameters. The validation dataset could result in fine-tuning the value of ω to reduce the weighted loss of the prediction model, as shown in Equation (26).

(26) $$\omega^{\prime }=\begin{array}{c} \min\\ \omega ,\omega^{\prime } \end{array}\frac{1}{m}\sum_{p=0}^{m-1}{f^{ʹ}}_{p}\left(\theta \;\times \;\omega \right)$$

To reduce negative training loss that could result in an unstable model, The value associated with the weight ω≥0 for all parameters p.

### 3.3. Dataset Description

The Pima Indian Dataset is used in the current study to predict Type-2 diabetes. It is part of the UCI machine learning repository maintained by the National Institute of Diabetes, Digestive, and Kidney Diseases. The dataset consists of eight columns representing parameters of Pregnancy, Glucose, Blood Pressure, Skin Thickness, Insulin, Body mass index (BMI), Diabetes Pedigree, and age. The PID dataset consists of a single output class with a binary value indicating whether or not an individual has diabetes. The dataset consists of 768 cases (500 non-diabetics and 268 diabetics) [72,73]. The Pima dataset is considered in the current study as it is widely used for comparing the performances of techniques. The dataset is partitioned as training and testing in a ratio of 70:30, with an initial learning rate of 0.0002, and it is observed that the model has

### 3.4. Implementation Environments

The computer is equipped with an Intel(R) Core i7(11th Gen) 4.70 GHz processor and 16 GB of main memory running over a 64-bit Windows 10 environment. The proposed RNN model for gene analysis is implemented over Kaggle, an online platform for executing such frameworks [74]. Python version 3.6.6, also widely known as the anaconda, is used in the implementation. Tensor Flow version 2.4.1, along with various libraries such as NumPy, pandas, matplotlib, seaborn, and sklearn, are used in the implementation process of the proposed model.

## 4. Results and Discussion

The proposed model has been evaluated on genomic data and the tabular data by using the same feature engineering mechanism and the layered approach for predicting the type-2 diabetes. The proposed RNN-based type-2 diabetes is evaluated against genomic and tabular data from the PIMA Indian dataset independently and the evaluations are presented independently in the current section. The model was evaluated against two datasets concerning various evaluation metrics such as sensitivity, specificity, Accuracy, and F1 score. The classification efficiency of the proposed model was assessed using true positive (TuP, the number of times that the model accurately predicted the gene with a high possibility of diabetes correctly), true negative (TuN, identifying the gene with less possibility of diabetes precisely), false positive (FsP, misinterpreting the gene with the high possibility of diabetes as low possibility of diabetes), and false negative (FsN, misinterpreting the low diabetes gene as a high possibility of illness). The sensitivity metric determines the ratio of how many were accurately recognized as positive samples out of how many were truly positive samples in the complete dataset. The specificity measure determines the ratio of how many were recognized as negative samples out of how many among the samples are truly negative from the complete dataset. The Accuracy measures the correctly predicted True positives and Negative samples against the overall sample in the complete dataset. The harmonic mean of sensitivity and specificity measures are determined as the F1 score. MCC is the best single-value classification score for summarizing the confusion matrix. The formulas for the aforementioned metrics are presented through Equations (27)–(32) [75].

(27) $$senstivity\left(recall\right)=\frac{TuP}{\left(TuP+FsN\right)}$$

(28) $$Specificity=\frac{TuN}{\left(TuN+FsP\right)}$$

(29) $$Accuracy=\frac{TuP+TuN}{\left(TuP+FsP+TuN+FsN\right)}$$

(30) $$Precision=\frac{Tup}{\left(TuP+FsP\right)\;}$$

(31) $$F1-score=2\times \frac{\;\left(precision\times recall\right)}{\left(precision+recall\right)}$$

(32) $$mcc=\frac{\left(TuP\times TuN\right)-\left(FsP\times FsN\right)}{\sqrt{\left(TuP+FsP\right)\left(TuP+FsN\right)\left(TuN+FsP\right)\left(TuN+FsN\right)}}$$

It is a far more appropriate statistical rate that yields a good score only if the prediction performed well among all the assumptions in the confusion matrix. The current section presents results about the experimental outcome of both genomic and tabular data with adequate analysis concerning existing models.

### 4.1. Experimental Outcome of Genomic Data

**Figure 7:**
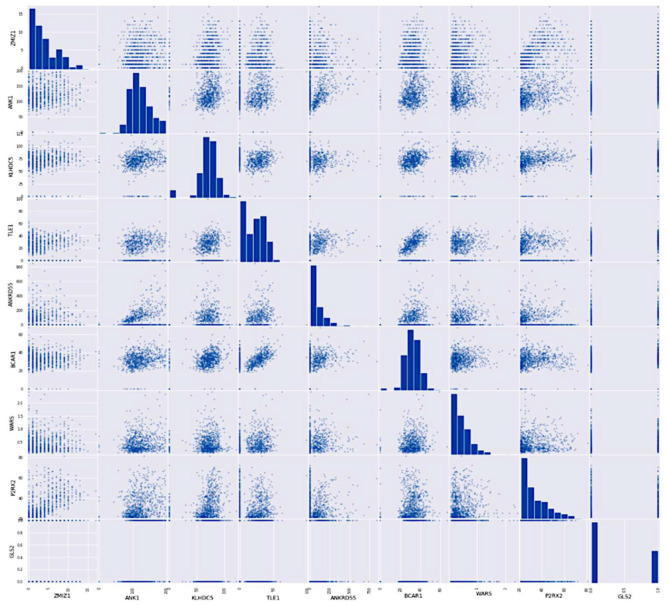
A scatter plot shows relationships among data points in input records.

The performance of the proposed RNN model for predicting type 2 diabetes was analyzed using performance evaluation metrics such as sensitivity, specificity, F1 score, Mathews correlation Coefficient, and accuracy measures [76]. The above-discussed metrics are assessed through true positive, true negative, false positive, and false negative values approximating experimental outcomes. The dataset is split into a training set and a validation set at a ratio of 70:30. In the following graph, as shown in Figure 7, it is clear that data values are skewed toward data instances, indicating that no diabetes exists. The percentage of available data records of non-diabetic patients (or those who do not have diabetes) is almost double that of diabetic patients.

**Figure 8:**
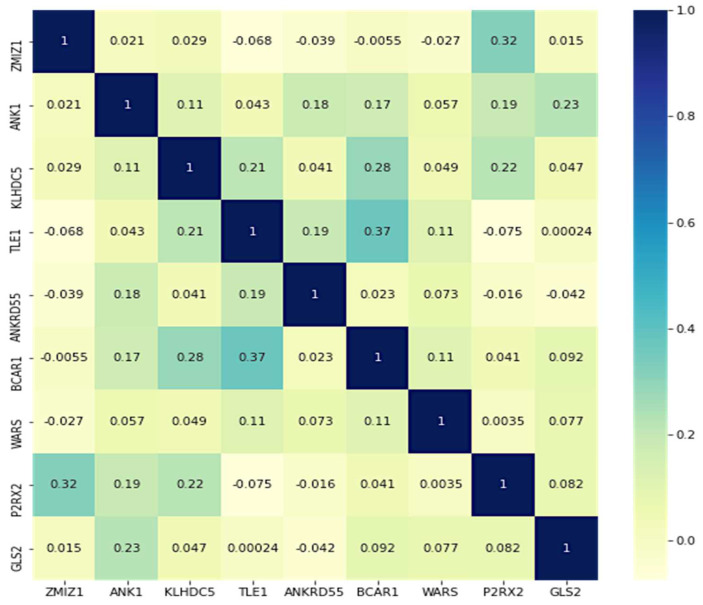
A heat map was generated from the gene dataset.

Correlation coefficients among data points as input gene data are analyzed using linear bivariate Pearson correlation coefficient (PCC). The correlation coefficient between two samples of gene expression is expressed as PCC. Correlation coefficient with a common confidence interval and covariance, the relationship among them is the ratio of the covariance of two variables and the product of their standard deviations. This gives a numeric representation of the covariance with an outcome between −1 and 1. Only a linear correlation between variables can be considered, even using the metric. Also, the metric does not represent several relationships or correlations. Figure 8 shows a two-dimensional heat map of data records. 

**Figure 9:**
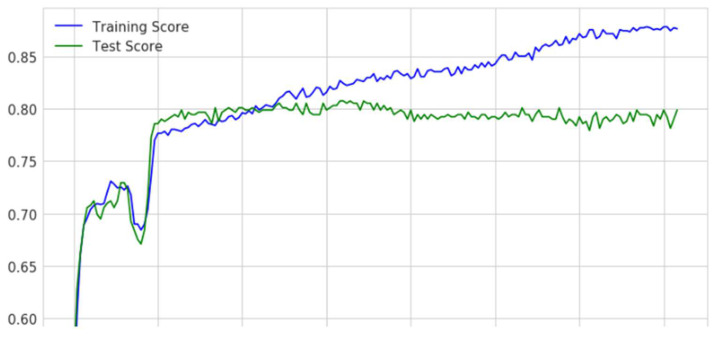
A graph showing training and testing scores of the proposed model.

Training and validation performances of the proposed model were evaluated using hyperparameters such as train and testing scores. The training score determined how perfectly the algorithm could generalize across its training samples. The testing score determined how well the model could accurately correlate the known gene sequence among individual records. An exceptionally high training score combined with a low-test result indicates overfitting. When the training score is quite low, and the test score is low, it indicates an underfitting. Performances of the proposed model concerning hyperparameters are presented in Figure 9.

**Figure 10:**
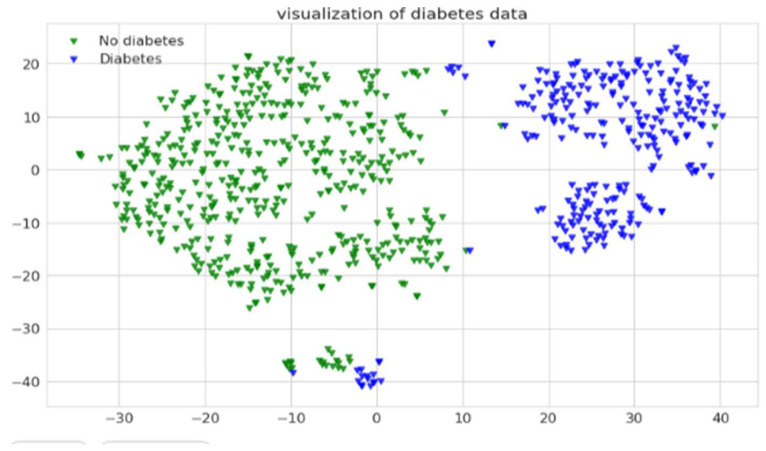
A graph showing the decision boundaries of two classes of records.

From graphs on the training and test scores, it can be depicted that the performance is reasonably fair in making the classifications precisely as there is no considerable deviation among either of the scores. In the current context, the gene expressions are classified as sequences with a high possibility of affecting type 2 diabetes and sequences with a low possibility of type 2 diabetes. The Decision Boundary is shown in a Scatter Plot, with every data point visualized on the data scatter plot and characteristics represented by x- and y-axes. The Decision Boundary forms a boundary for dividing data points into regions and their classes. Categories of gene sequences with high and low possibilities of developing diabetes are shown in Figure 10.

**Figure 11:**
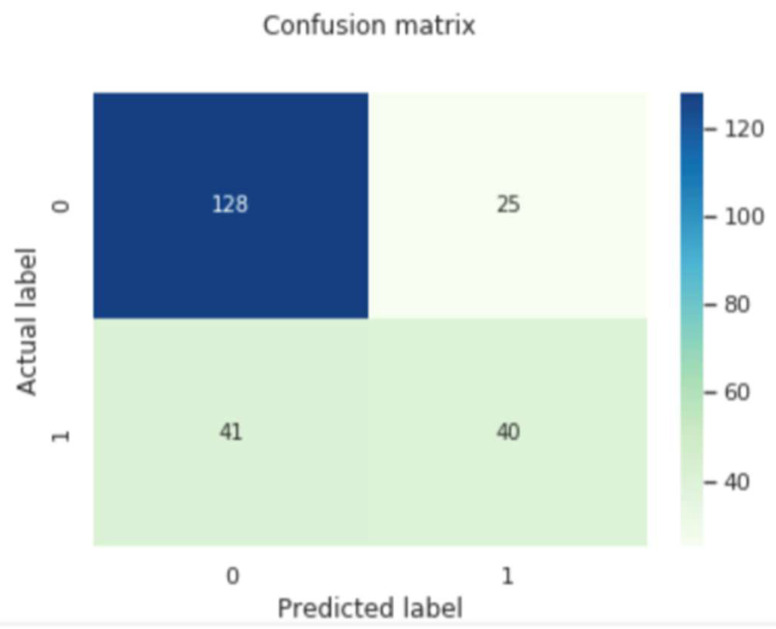
Image representing the confusion matrix for the proposed RNN model for future illness prediction.

The confusion matrix would assist in analyzing the performance of the proposed model in analyzing future illness. The evaluated samples, i.e., TuP, TuN, FsP and FnP are shown in the confusion matrix in Figure 11, and the corresponding performance evaluation metrics are shown in Table 4.

**Figure 12:**
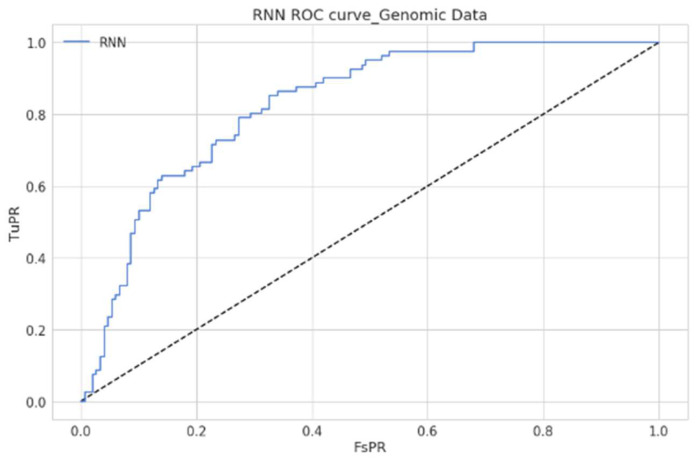
Graph presenting the ROC curve of the proposed model.

As shown in Table 4, estimated values clearly demonstrated that the model made predictions reasonably with few records. However, the model’s performance could be further improvised when more data records could be used. The Receiver Operating Characteristic (RoC) Curve of the proposed model is presented in Figure 12, and it is depicted that the model has outperformed with reasonable accuracy in precisely classifying the genomic data. The RoC curve estimates how well the proposed approach can differentiate the two-class records that include gene expression with a higher or lower possibility of being affected with type-2 diabetes. An accurate model can tell the difference between the two. An improper model will find it difficult to tell the difference between the two sets of records.

### 4.2. Experimental Outcome with Tabular Data (PIMA Dataset)

**Figure 13:**
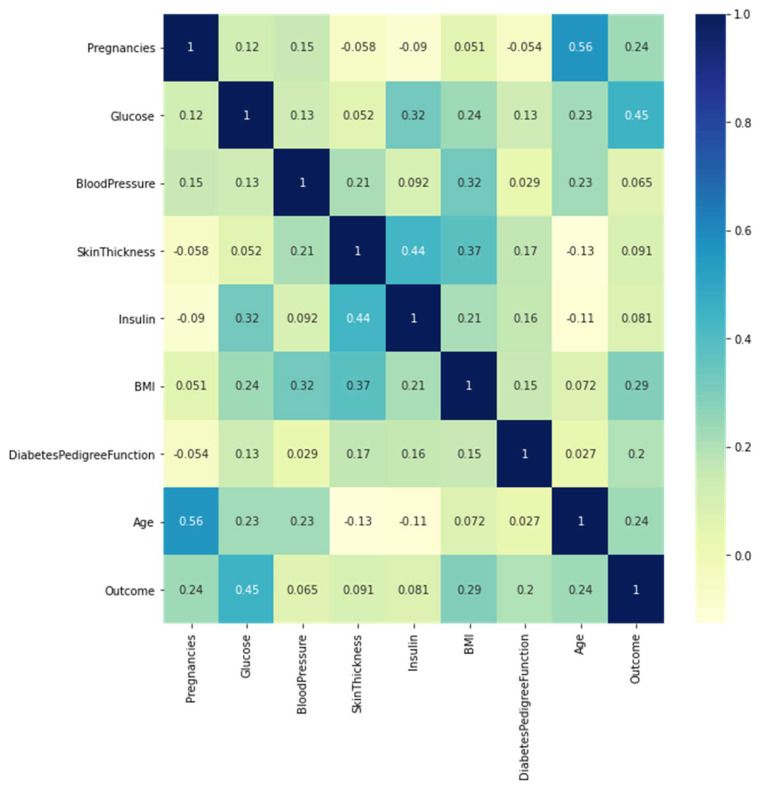
Heat map generated with the PIMA dataset.

The Pima Indian dataset consists of eight features that help predict the possibility of affecting type-2 diabetes. The model’s performance was evaluated using various evaluation metrics. The heat map represents the association of multiple parameters in determining a future illness. Figure 13 illustrates the heat map of features in the PIMA Indian dataset. 

**Figure 14:**
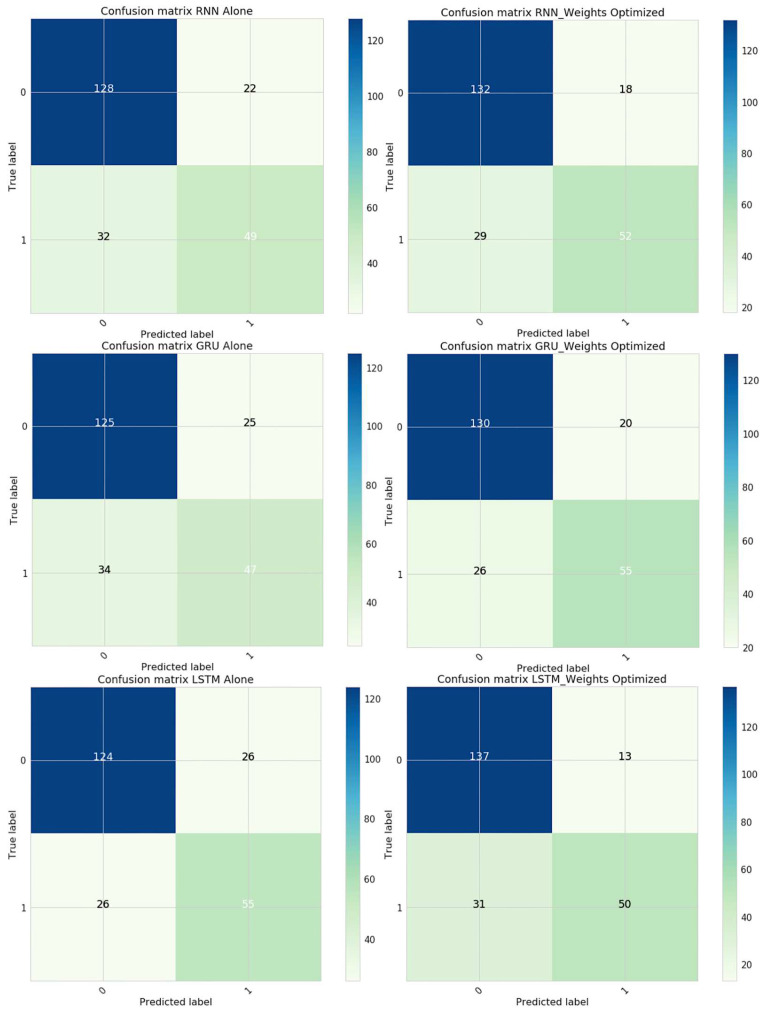
Confusion matrix of the proposed model.

The experimentation was performed with the PIMA data by optimizing initial weights assigned to parameters. The proposed model exhibited better accuracy in optimizing weights. Figure 14 presents the resultant confusion matrix obtained over data with and without weight optimization for all three recurrent neural network components. Among these, the LSTM-based architecture has outperformed in terms of classification accuracy. Correctly identifying the individual record as a diabetic patient was assumed as a True Positive (TuPR). The correctly predicting the non-diabetic patient was assumed as a True Negative (TuNR). When the model misinterpreted normal cases as diabetic cases, False Positive (FsPR) was considered. When diabetic cases were recognized as normal cases, False Negative (FsNR) was considered.

The percentage of true positives that are accurately recognized is what sensitivity analyzes. Specificity, often known as the real negative rate, is a measurement that determines the percentage of actual negative instances that are accurately classified as such. The ratio of the number of instances properly categorized to the total number of instances is called Accuracy. The F1 score is a statistic calculated by taking the harmonic mean of a classifier’s accuracy and recall values and combining them into a single value. A low number of false positives and false negatives gives you an excellent F1 score. The Matthews correlation coefficient, or MCC, is a correlation coefficient that compares predicted values to actual values, which is mostly used in binary class classification problems. The weight optimization process could help evaluate the dataset more precisely as features with more significance would be considered in the evaluation process. Weights are optimized over the iteration. Resultantly, more significant features are involved in the evaluation process. The weight optimization could yield considerable Accuracy over a conventional model. The experimental outcome presented in Table 5 shows the outcome of the proposed model concerning optimized weights.

The classification efficiency assessment of the proposed model was compared with various existing studies concerning evaluation parameters such as sensitivity, specificity, Accuracy, and F1 score. Table 6 presents experimental values obtained by the proposed model over other existing models such as Naive Bayes, J48, Logistic Regression, K Nearest Neighbor, Random Forest, Decision Tree, REPTree, Sequential Minimal Optimization (SMO) and BayesNet. Experimental outcomes of the current model are evaluated against the outcomes of other existing models using similar datasets [77,78].

A resampling technique for evaluating the machine learning approaches is known as cross-validation, where a small data sample is considered for evaluation. The technique includes a single parameter, k which specifies how many groups are provided with sample data. The k-fold cross-validation describes the number of groups associated with the evaluation. When *k* = 2 means the model reference to 2-fold cross-validation. The formula for the cross-validation over kf folds concerning to the mean square error (MSE) is shown in Equation (33). In the current study, the accuracies of the RNN model with different auxiliary memory components are evaluated against divergent K-Values, as presented in Table 7.

(33) $$cross\_validation_{k_{f}}=\frac{1}{k_{f}}\sum_{x=1}^{k_{f}}mse_{x}$$

**Figure 15:**
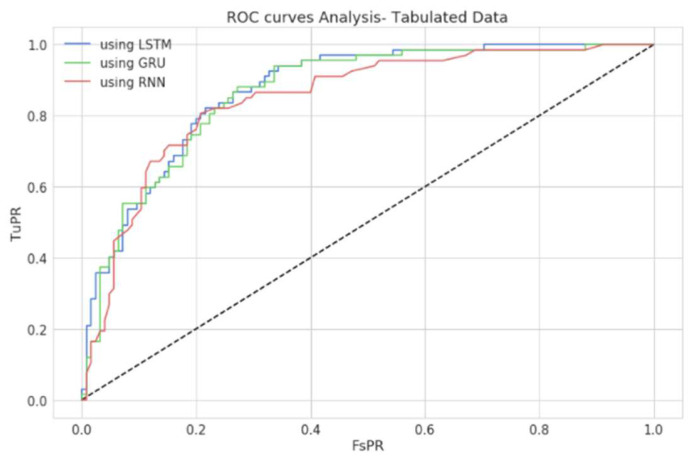
ROC curve of the proposed model using PIMA data.

The ROC curve of the proposed model states the trade-off between the True Positive assumption and the False Positive assumption of the proposed model concerning predictions made with the Pima diabetic dataset. Figure 15 presents the ROC of the proposed model with optimized weights using tabular data. The proposed model has shown the classification’s desired performance concerns.

The present model was trained with limited genomic data or PIMA diabetic dataset. Either of these datasets consisted of approximately 700 records. Of them, 30% of the overall data were meant for testing, resulting in training the model with inadequate records that could impact its performance. RNN-based models in various applications have exhibited noticeable accuracies. However, the neural network model’s Accuracy can vary depending on the ratio of the training sample to the testing sample.

### 4.3. Practical Implications

The proposed technique for forecasting type-2 diabetes can be implemented over a mobile framework with a front-end module. Patients and practitioners can perform the initial assessment of the illness. Users can provide details such as glucose levels, pregnancies, insulin, hypertension, BMI, Diabetes Pedigree, skin thickness, and heart rate. Based on the provided input and the trained data, the model can analyze the input with the trained data for predicting the illness. The model can be implemented in the iOS platform to the back-end Kaggle using the back-end service such as the MBaaS component. A secured socket layer (SSL) and two-factor authentication can ensure the security of the model [79,80].

**Figure 16:**
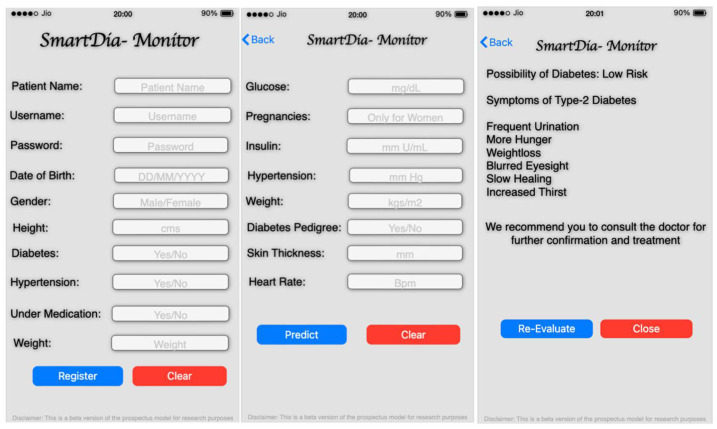
Images presenting the mobile interface of the future implication model.

Images of the user interface of the application model are presented in Figure 16. The leftmost image represents the registration page of the application. The middle image shows the user information page, followed by the resultant prediction screen of the model. The model makes the task of predicting a future illness more convenient. The model can be improved by incorporating the genomic module for accepting gene data and evaluating the illness based on gene information.

The future implication model was inspired by a non-invasive way of assessing future illness more precisely. The assessment could help physicians and individuals better evaluation of health conditions. The prediction could assist individuals in adopting better life standards and living habits to avoid or prolong the chance of being affected by the illness [81]. The computationally efficient approaches like the MobileNet V2 and MobileNet V3 architectures would assist better in deploying the models in lightweight computational devices. The RNN models need tremendous computation efforts despite providing highly accurate performances.

## 5. Conclusions

The genomic-based future illness prediction is a path-breaking approach for precisely assessing future illness. Genomic-based data can be conveniently analyzed through supervised-based approaches such as Neural Network models. The sequence of GENE can be downsampled and analyzed based on the weight concerning the diseased sequence. The approach decomposes a large GENE sequence into smaller GENE strings, raising the chances of accurate matching with diseased sequences and resulting in a precise prediction. Although there might be a considerable burden on the machine to decompose the large genomic patterns, decomposing them to a certain predetermined extent has better Accuracy than conventional approaches. When the proposed model was evaluated over the PIMA diabetic dataset, it exhibited a reasonable performance in predicting type-2 diabetes. The PIMA dataset consists of 768 records, of which only 537 are used for training. The Accuracy would be much better when more records are for training purposes. Statistical analysis for disease progression [82] has exhibited better performance than the existing models when adequate data are available. Users may access the suggested model’s prediction result via the Android application. As a result, it is desired to give an effective method for determining the possibility of being affected by diabetes at an early stage. However, it is exceedingly challenging to work with larger sequence gene data, the quantum and federated learning techniques would effectively handle such a larger sequence data. On the other side, when dealing with tabular data, the ensemble classification models would yield almost identical performance with minimal computation to the suggested RNN models.

Although the proposed approach showed promising results, it was challenging when decomposing to a more significant extent. In such situations, incorporating Long Short-Term Memory (LSTM) can make the approach more robust with considerably lesser computational latency. For handling an unusual illness, self-Learning Based algorithms and the use of cognitive technology would be appropriate to minimize the steps needed for training the algorithm [83]. The proposed approach based on Genomics with Self-Learning algorithms might result in better results than supervisory approaches alone. In future work, comparison with other smart diagnosis techniques and assessment of other clinical datasets need to be performed. Once the model validation is performed with more datasets, other risk factors affecting diabetes can be revealed. The future dimensions of the research include the deep learning-driven pattern recognition models for analyzing the gene sequences for identifying the possible future illness and developing mobile applications that can generalize the information from the genomic data. However, there is great demand for explainable Artificial Intelligence models that are interpretable in decision-making.

## Figures and Tables

**Table 1:** Various existing models for diabetes prediction.

Approach	Type of Data	Applicability	Limitations
polygenic scores-based approach [12]	Genomic Data	Used in the evaluation of clinical trials and illness screening mechanisms	The polygenic score approach needs larger samples and tremendous training for considerable Accuracy.
Singular Value Decomposition [13]	Genomic Data Tabular Data The image they are used	They are used in ranking the feature set and compression of the data through the least-square fitting. Gene sequences are ranked based on the probability of illness.	SVD is not an algorithm designed to perform; it is a matrix decomposition mechanism. They are various neural ranking models that perform much better than SVD.
Principle Component Analysis [14]	Genomic Data Tabular Data	PCA technique is extensively used in gene analysis to discover the regional and ethnic patterns of genetic variation.	The independent gene expressions are less interpretable, and information loss is possible if the number of components is carefully chosen.
Gene Co-Expression model [27]	Genomic Data	The Gene Co-Expression model analyzes the genomic data’s insights through similarity assessment of expressions and topologies.	The Gene Co-Expression model may not deal with larger features than the data size and non-linearity in the network architecture.
Reinforcement approaches (SARSA, DDPG, DQN) [28]	Genomic Data Tabular Data Image Data	The reinforcement learning models are widely used in studies where the states in the problem are deterministic and in situations where control over the environment is needed. RL models are proven to exhibit better non-linearity in gene analysis.	Adding excessive amounts of reinforcement learning may result in an overflow of states, which might reduce the effectiveness of the findings. As well, RL models are data-hungry.
Decision Tree [39]	Tabular Data Image Data	Using Decision Trees, the efforts to preprocess data can be reduced as normalization and scaling are not required, and missing values will not influence the model’s outcome.	DT models consume more time to train the model, and more effort is desired.
J48 [40]	Tabular Data Image Data	J48 is a decision tree that can handle outliers effectively and robustly in non-linear problems.	J48 model is less stable, and noisy data compromises the efficiency of the data.
K Nearest Neighbor [41]	Tabular Data Image Data	The K Nearest Neighbor model does not need prior training for classifying the class data. It requires lesser computational efforts and a faster resultant outcome.	The KNN model fails to work with a larger dataset and high-dimensional data. The feature scaling phase is crucial for an optimal classification level, which requires considerable effort.
Logistic Regression [42]	Tabular Data Image Data	Logistic Regression is the very predominantly used classification technique. The model efficiently classifies the data based on the likelihood and the association among the data items. The model can sustain the overfitting and underfitting issues.	The challenging part of the Logistic Regression is linear separatable and often leads to overfitting when observations are fewer concerning the feature set size.
Naive Bayes [43]	Tabular Data Image Data	Naive Bayes algorithms perform well for multi-class classification models with minimal training.	NB assumes all the feature vectors as mutually independent components in the classification process. NB may not perform better in evaluating the problems with the interdependent feature set.
Random Forest [44]	Tabular Data Image Data	Random Forest models perform bagging for classification. RF models efficiently reduce the over-fitting issue and can handle the missing effectively. Moreover, the feature scaling task need not be performed.	RF models need tremendous training, and frequent hyperparameter tuning is required for considerable Accuracy.
Support Vector Machine [45]	Tabular Data Image Data	Support Vector Machine is efficient in handling thigh-dimensional and efficient memory handling capability.	SVM is inappropriate for working with a larger dataset with a larger feature set. The outcome of the SVM model is largely dependent on the objective function. Too many support vectors will be generated when choosing a larger kernel, which might impact the model’s training process.
Genetic Algorithm [46]	Genomic Data Tabular DataImage Data	A genetic algorithm is an evolutionary algorithm that uses probabilistic transaction rules, and non-linearity in the searching process would yield better model accuracy. As well, can effectively handle the larger search space.	The genetic algorithm has susceptible to local maxima and minima and similarly to global maxima and minima. That might result in poor prediction performances.

**Table 2:** Genomic information associated with Type 2 diabetes.

Gene Data	Type 2 Diabetes	Fasting Glucose	Alleles	SNP	Megabase
GLS2		✔	G/A	rs2657879	55.2
P2RX2		✔	A/G	rs10747083	131.6
WARS		✔	G/T	rs3783347	99.9
BCAR1	✔		T/G	rs7202877	73.8
ANKRD55	✔		G/A	rs459193	55.8
TLE1	✔		G/A	rs2796441	83.5
KLHDC5	✔		C/T	rs10842994	27.9
ANK1	✔		C/T	rs516946	41.6
ZMIZ1	✔		A/G	rs12571751	80.6

**Table 3:** Feature set associated with Type 2 diabetes.

Feature	Data_Type	Min_Value	Max_Value	Information Gain	Mean Rank
Glucose (mg/dL)	Integer	0	199	0.2497	3
Pregnancies	Integer	0	17	~	~
Age	Integer	21	81	0.0761	3.17
Heart Rate	Integer				7.67
Waist	Integer	~	~	0.0356	9.5
Pulse Pressure	Integer			~	12.33
Insulin (mm U/mL)	Integer	0	846	~	13.33
Hypertension (Blood Pressure) (mm Hg)	Integer	0	122	0.0304 (bp1), 0 (bp2)	15
BMI (weight) (kg/m^2^)	Real	0	67.1	~	~
Diabetes Pedigree Function	Real	0.08	2.42	~	~
Skin thickness (mm)	Real	0	99	~	~

**Table 4:** Performance evaluation metric and estimated values.

Metric	Estimated Value
Sensitivity	83.66
Specificity	49.38
Precision	75.73
Accuracy	71.79
Mathew’s correlation Coefficient	35.09

**Table 5:** Performance of the proposed model with weight optimization.

	Sensitivity	Specificity	Accuracy	F1-Score	MCC
RNN Model	0.800	0.690	0.753	0.825	0.473
RNN + GRU	0.786	0.652	0.744	0.809	0.426
RNN + LSTM	0.826	0.679	0.774	0.823	0.505
RNN Model (WO)	0.819	0.742	0.796	0.848	0.541
RNN + GRU(WO)	0.833	0.733	0.800	0.849	0.558
RNN + LSTM(WO)	0.815	0.793	0.810	0.856	0.568

**Table 6:** Performance analysis of the proposed model with existing studies.

	Sensitivity	Specificity	Accuracy	F1-Score	MCC
Decision Tree	0.781	0.561	0.697	0.762	0.349
J48	0.688	0.695	0.691	0.754	0.383
K Nearest Neighbour	0.748	0.603	0.708	0.787	0.331
Logistic Regression	0.775	0.666	0.744	0.813	0.416
Naive Bayes	0.820	0.687	0.689	0.830	0.502
Random Forest	0.789	0.661	0.750	0.813	0.436
Support Vector Machine	0.775	0.666	0.744	0.813	0.416
REPTree	0.530		0.744	0.590	
SMO	0.280		0.724	0.410	
BayesNet	0.570		0.738	0.600	
RNN model	0.837	0.774	0.818	0.864	0.591

**Table 7:** The Accuracy of the RNN model with auxiliary memory components against divergent K- Values.

Value of K	RNN Model	RNN + GRU	RNN + LSTM	RNN Model (WO)	RNN + GRU (WO)	RNN + LSTM (WO)
2	0.716	0.704	0.723	0.752	0.771	0.789
5	0.745	0.739	0.770	0.791	0.799	0.812
10	0.774	0.762	0.798	0.810	0.821	0.824

## Data Availability

Not applicable.
